# Supplementary material for: Digital non-Foster-inspired electronics for broadband impedance matching
Source: Nat Commun. 2024 May 21;15:4346. doi: 10.1038/s41467-024-48861-6 (PMC11109259; doi:10.1038/s41467-024-48861-6)
Supplement: Supplementary file 1 — Supplementary Information [file 41467_2024_48861_MOESM1_ESM.pdf]

Supplementary Information

Digital Non-Foster-Inspired Electronics for Broadband Impedance Matching

Xin Yang[1,2]†*, Zhihe Zhang[1]†, Mengwei Xu[1], Shuxun Li[1], Yuanhong Zhang[1], Xue-Feng Zhu[3*], Xiaoping Ouyang[4*], Andrea Alù[5*]

[1]*College of Electrical and Information Engineering, Hunan University, Changsha 410082, People's Republic of China.*

[2]*Engineering Research Center of Advanced Semiconductor Technology and Application of Ministry of Education, Hunan University, Changsha 410082, People's Republic of China.*

[3]*School of Physics, Huazhong University of Science and Technology, Wuhan, Hubei 430074, People's Republic of China.*

[4]*School of Materials Science and Engineering, Xiangtan University, Xiangtan 411105, People's Republic of China.*

[5]*Photonics Initiative, Advanced Science Research Center, City University of New York, New York, New York 10031, USA*

Corresponding Authors: Xin Yang[1,2], Xue-Feng Zhu[3*], Xiaoping Ouyang[4*], Andrea Alù[5*]

*E-mails: xyang@hnu.edu.cn, xfzhu@hust.edu.cn, oyxp2003@aliyun.com, aalu@gc.cuny.edu

†These authors contributed equally to this work.

Supplementary Notes

Supplementary Note 1: Impedance modeling and bandwidth estimation of the electroacoustic transducer.

The radiated acoustic power can be calculated by analysis of the analogous circuit to yield the transducer bandwidth[1]. The analogous circuit for a common electroacoustic transducer[2] is shown in Fig. 2**a** of main text. For the electrical part, R_{E} is the DC voice-coil resistance. $L_{\mathrm{E}}(\omega)$ is the voice-coil inductance while $R'_{\mathrm{E}}(\omega)$ is the voice-coil resistance caused by eddy currents, both of which have certain FD[3]. u_{E} is the input voltage of the transducer, and the input of the electrical-mechanical gyrator u_{e} reflects the voltage induced when the voice coil vibrates with a speed of v_{D}. Here, the voice-coil current i_{E} is

$$i_{\mathrm{E}} = \frac{u_{\mathrm{E}} - u_{\mathrm{e}}}{R_{\mathrm{E}} + Z'_{\mathrm{E}}(\omega)} = \frac{u_{\mathrm{E}}}{R_{\mathrm{E}} + Z'_{\mathrm{E}}(\omega)} - \frac{Blv_{\mathrm{D}}}{R_{\mathrm{E}} + Z'_{\mathrm{E}}(\omega)}, \quad (1)$$

where Bl is the force factor, in unit of N·A^{-1}.

v_{D} can be solved by

$$v_{\mathrm{D}} = \frac{f_{\mathrm{D}} - f_{\mathrm{a}}}{Z_{\mathrm{m}}}, \quad (2)$$

where f_{D} is the force exerted on the diaphragm and f_{a} is the force generated by the acoustic pressure difference p_{D} (in unit of Pa) across the diaphragm. They can be derived as

$$\begin{cases} f_{\mathrm{D}} = Bli_{\mathrm{E}} \\ f_a = S_{\mathrm{D}} p_{\mathrm{D}} = S_{\mathrm{D}} V_{\mathrm{D}} Z_{\mathrm{rad}} \end{cases}, \quad (3)$$

where S_{D} is the area of the diaphragm. V_{D} represents the volume velocity emitted by the diaphragm in unit of m^3·s^{-1} and Z_{rad} is the acoustic radiation impedance in unit of Pa·s·m^{-3}.

The mechanical impedance Z_{m} in unit of kg·s^{-1} can be expressed by

$$Z_{\mathrm{m}} = R_{\mathrm{MS}} + j\omega M_{\mathrm{MD}} + \frac{1}{j\omega C_{\mathrm{MS}}}, \quad (4)$$

where M_{MD} is the mechanical mass of the diaphragm and voice-coil assembly, C_{MS} is the mechanical compliance of the diaphragm suspension in unit of m·N^{-1} and R_{MS} is the mechanical loss in the suspension in unit of N·s·m^{-1}.

Eventually, the input-output relationship between u_{E} and V_{D} can be obtained as

$$V_{\mathrm{D}}\left[\frac{(Bl)^2}{S_{\mathrm{D}}^2\left(R_{\mathrm{E}}+Z_{\mathrm{E}}'(\omega)\right)}+\frac{Z_{\mathrm{m}}}{S_{\mathrm{D}}^2}+Z_{\mathrm{rad}}\right]=\frac{Blu_{\mathrm{E}}}{S_{\mathrm{D}}\left(R_{\mathrm{E}}+Z_{\mathrm{E}}'(\omega)\right)}. \quad (5)$$

when the transducer is loaded with the digital non-Foster-inspired circuit, **Supplementary** Eq. (5) will be rewritten as Eq. (2). Over the low frequency range, the transducer is conceived to operate as a rigid piston mounted in a flat infinite baffle board[4], which can be treated as an acoustic monopole model[2,4]. The analogous circuits are widely used to estimate the basic vibration and radiation characteristics of transducers, and Z_{rad} on a piston in an infinite baffle can be calculated by **Supplementary** Eq. (6) as follows[2]

$$Z_{\mathrm{rad}}=\frac{-\omega^2 C_{\mathrm{A1}}R_{\mathrm{A1}}R_{\mathrm{A2}}M_{\mathrm{A1}}+j\omega M_{\mathrm{A1}}\left(R_{\mathrm{A1}}+R_{\mathrm{A2}}\right)}{R_{\mathrm{A1}}+R_{\mathrm{A2}}-\omega^2 C_{\mathrm{A1}}R_{\mathrm{A1}}M_{\mathrm{A1}}-j\omega\left(C_{\mathrm{A1}}R_{\mathrm{A1}}R_{\mathrm{A2}}+M_{\mathrm{A1}}\right)}, \quad (6)$$

The acoustical elements R_{A1}, R_{A2} (in unit of N·s·m^{-5}), C_{A1} (in unit of m^5·N^{-1}) and M_{A1} (in unit of kg·m^{-4}) are respectively

$$\begin{cases} M_{\mathrm{A1}}=\dfrac{16\rho_0}{3\pi^2 a}, & R_{\mathrm{A1}}=\dfrac{0.8820\rho_0 c}{\pi a^2} \\ R_{\mathrm{A2}}=\dfrac{2\rho_0 c}{\pi a^2}, & C_{\mathrm{A1}}=\dfrac{2.97a^3}{\rho_0 c^2} \end{cases}, \quad (7)$$

a is piston radius of the electroacoustic transducer. ρ_0 and c_0 are air density and sound velocity in air, respectively.

Eventually, the analogous circuit can be modeled, as shown in **Supplementary** Fig. 1**a**. The obtained theoretical impedance is consistent with the measured results extracted by the power analyzer (PW6001, HIOKI), as shown in **Supplementary** Figs. 1**b** and **c**.

Based on the above analysis, the acoustic power P_{ar} of the transducer (in unit of W) with our proposed circuit can be calculated by Eq. (1). According to the half-power bandwidth definition[1], the

frequency corresponding to the maximum acoustic power P_{m} needs to be acquired by

$$\frac{\mathrm{d}P_{\mathrm{ar}}(f, R_{\mathrm{D}}, L_{\mathrm{D}})}{\mathrm{d}f} = 0 , \tag{8}$$

which, however, cannot be solved directly without knowing R_{D} and L_{D}, much less $R_{\mathrm{D}}(\omega)$ and $L_{\mathrm{D}}(\omega)$ with FD. Thus, the values of f_{l} and f_{h} at the half-power points $P_{\mathrm{m}}/2$ cannot further be mathematically derived. For this reason, the exhaustion method is applied to sweep over R_{D} and L_{D} for calculating the bandwidth $\Delta f = f_{\mathrm{h}} - f_{\mathrm{l}}$ as shown in Fig. 2**b** of the main text, where the maximum bandwidth point p_2 (R_D, L_D) and p_4 (0, L_D) are marked. $Z'_{\mathrm{E}}(\omega)$ with FD is the main component of the transducer impedance at frequencies far from the resonance frequency. It severely limits the operation bandwidth. The system bandwidths by impedance matching configurations p_1 ($R_{\mathrm{D}}(\omega)$, $L_{\mathrm{D}}(\omega)$) and p_3 (0, $L_{\mathrm{D}}(\omega)$) are further derived and also marked in Fig. 2**b** of the main text.

Supplementary Note 2: Stability analysis. Although a large gain can guarantee the steady-state response of the system and small steady-state errors, attention must be paid to the transient response[5]. The system is unstable if any pole of the system transfer function moves to the right half of the complex plane. So as to analyze the stability of loading the electroacoustic transducer with the digital non-Foster-inspired electronics, the general circuit structure shown in Fig. 3**a** is simplified as the transfer function block diagram (**Supplementary** Fig. 2**a**)[6]. Further, according to Mason's gain formula, the block function can be derived in **Supplementary** Fig. 2**b** as

$$\begin{aligned} I_{\mathrm{o}}(s) &= G_{\mathrm{Iref}}(s) I_{\mathrm{ref}}(s) + G_{\mathrm{Us}}(s) U_{\mathrm{s}}(s) \\ &= \frac{(P_1\Delta_1 + P_2\Delta_2)\cdot I_{\mathrm{ref}}(s) + P_3\Delta_3 \cdot U_{\mathrm{s}}(s)}{1 - L_1 - L_2 - L_3} \end{aligned} \tag{9}$$

where

$$\begin{cases} P_1 = -\dfrac{G_{\mathrm{PR}}(s)G_{\mathrm{PWM}}(s)}{s^2L_{\mathrm{s}}C_{\mathrm{s}}(R_{\mathrm{T}}+sL_{\mathrm{T}})} \\ P_2 = -\dfrac{K_{\mathrm{f}}G_{\mathrm{PWM}}(s)}{s^2L_{\mathrm{s}}C_{\mathrm{s}}(R_{\mathrm{T}}+sL_{\mathrm{T}})} \\ P_3 = \dfrac{1}{(R_{\mathrm{T}}+sL_{\mathrm{T}})} \end{cases}, \quad \begin{cases} L_1 = \dfrac{G_{\mathrm{PR}}(s)G_{\mathrm{PWM}}(s)}{s^2L_{\mathrm{s}}C_{\mathrm{s}}(R_{\mathrm{T}}+sL_{\mathrm{T}})} \\ L_2 = -\dfrac{1}{sC_{\mathrm{s}}(R_{\mathrm{T}}+sL_{\mathrm{T}})} \\ L_3 = -\dfrac{1}{s^2L_{\mathrm{s}}C_{\mathrm{s}}} \end{cases}, \quad \begin{cases} \Delta_1 = 1 \\ \Delta_2 = 1 \\ \Delta_3 = 1-L_3 \end{cases} \tag{10}$$

where R_{T} and L_{T} denote the equivalent resistance and inductance of the electroacoustic transducer, respectively. A feedforward gain K_{f} is often added to handle the conflict between steady-state response, transient response, and stability[7]. Additionally, the self-adaptive PR controller is the software core of our proposed circuit. The transfer function of the PR controller is introduced[8]

$$G_{\mathrm{PR}}(s) = K_{\mathrm{p}} + K_{\mathrm{r}}\frac{2\omega_{\mathrm{c}}s}{s^2+2\omega_{\mathrm{c}}s+\omega_0^2}, \tag{11}$$

where ω_{c} represents the cutoff bandwidth around the resonant frequency ω_0. Also, K_{p} and K_{r} are the proportional term and the integral term of the controller, respectively. K_{p} ensures good transient performance and stability, while K_{r} can eliminate the amplitude and phase steady-state errors[5].

Moreover, $G_{\mathrm{PWM}}(s)$ can be modeled as[9]

$$G_{\mathrm{PWM}}(s) = \frac{1-0.5T_{\mathrm{s}}s}{(1+0.5T_{\mathrm{s}}s)^2}. \tag{12}$$

Supplementary Fig. 2**c** illustrates the corresponding open-loop transfer function block diagram. By simplifying the open-loop control block diagram, and the transfer function from I_{ref} to I_{o} can be derived as

$$G_{\mathrm{open}}(s) = -\frac{G_{\mathrm{PR}}(s)G_{\mathrm{PWM}}(s)+K_{\mathrm{f}}G_{\mathrm{PWM}}(s)}{s^3L_{\mathrm{s}}C_{\mathrm{s}}L_{\mathrm{T}}+s^2L_{\mathrm{s}}C_{\mathrm{s}}R_{\mathrm{T}}+sL_{\mathrm{T}}+sL_{\mathrm{s}}+R_{\mathrm{T}}+K_{\mathrm{f}}G_{\mathrm{PWM}}(s)} \tag{13}$$

By evaluating the poles' locations of the closed-loop transfer function as K_{p}, K_{r}, K_{f}, and ω_{c} vary, we can observe what parameter ranges can assure the system stability. According to the above equations, the characteristic equation $D(s)=0$ of system can be derived.

$$D(s) = a_0s^7+a_1s^6+a_2s^5+a_3s^4+a_4s^3+a_5s^2+a_6s+a_7=0, \tag{14}$$

where coefficients a_i (i=0, 1, 2, 3, 4, 5, 6, 7) are respectively

$$
\begin{aligned}
&a_0 = C_\mathrm{s} L_2 L_\mathrm{s} T_\mathrm{s}^2 \\
&a_1 = 4C_\mathrm{s} L_2 L_\mathrm{s} T_\mathrm{s} + C_\mathrm{s} L_\mathrm{s} R_2 T_\mathrm{s}^2 + 2C_\mathrm{s} L_2 L_\mathrm{s} T_\mathrm{s}^2 \omega_\mathrm{c} \\
&a_2 = L_2 T_\mathrm{s}^2 + L_\mathrm{s} T_\mathrm{s}^2 + 4C_\mathrm{s} L_2 L_\mathrm{s} + 4C_\mathrm{s} L_\mathrm{s} R_2 T_\mathrm{s} + 2C_\mathrm{s} L_\mathrm{s} R_2 T_\mathrm{s}^2 \omega_\mathrm{c} + C_\mathrm{s} L_2 L_\mathrm{s} T_\mathrm{s}^2 \omega_0^2 + 8C_\mathrm{s} L_2 L_\mathrm{s} T_\mathrm{s} \omega_\mathrm{c} \\
&a_3 = R_2 T_\mathrm{s}^2 + 4L_2 T_\mathrm{s} + 4L_\mathrm{s} T_\mathrm{s} + 4C_\mathrm{s} L_\mathrm{s} R_2 + 2L_2 T_\mathrm{s}^2 \omega_\mathrm{s} + 2L_\mathrm{s} T_\mathrm{s}^2 \omega_\mathrm{c} + 8C_\mathrm{s} L_2 L_\mathrm{s} \omega_\mathrm{c} + 4C_\mathrm{s} L_2 L_\mathrm{s} T_\mathrm{s} \omega_0^2 \\
&\quad + C_\mathrm{s} L_\mathrm{s} R_2 T_\mathrm{s}^2 \omega_0^2 + 8C_\mathrm{s} L_\mathrm{s} R_2 T_\mathrm{s} \omega_\mathrm{c} \\
&a_4 = 4L_2 + 4L_\mathrm{s} + 4R_2 T_\mathrm{s} + 2T_\mathrm{s} K_\mathrm{p} + L_2 T_\mathrm{s}^2 \omega_0^2 + L_\mathrm{s} T_\mathrm{s}^2 \omega_0^2 + 8L_2 T_\mathrm{s} \omega_\mathrm{c} + 8L_\mathrm{s} T_\mathrm{s} \omega_\mathrm{c} + 2R_2 T_\mathrm{s}^2 \omega_\mathrm{c} + 8C_\mathrm{s} L_\mathrm{s} R_2 \omega_\mathrm{c} \,. \qquad (15) \\
&\quad + 4C_\mathrm{s} L_2 L_\mathrm{s} \omega_0^2 + 4C_\mathrm{s} L_\mathrm{s} R_2 T_\mathrm{s} \omega_0^2 \\
&a_5 = 4R_2 - 4K_\mathrm{p} + 8L_2 \omega_\mathrm{c} + 8L_\mathrm{s} \omega_\mathrm{c} + R_2 T_\mathrm{s}^2 \omega_0^2 + 8R_2 T_\mathrm{s} \omega_\mathrm{c} + 4T_\mathrm{s} K_\mathrm{i} \omega_\mathrm{c} + 4T_\mathrm{s} K_\mathrm{p} \omega_\mathrm{c} + 4L_2 T_\mathrm{s} \omega_0^2 \\
&\quad + 4L_\mathrm{s} T_\mathrm{s} \omega_0^2 + 4C_\mathrm{s} L_\mathrm{s} R_2 \omega_0^2 \\
&a_6 = 8R_2 \omega_\mathrm{c} - 8K_\mathrm{i} \omega_\mathrm{c} - 8K_\mathrm{p} \omega_\mathrm{c} + 4L_2 \omega_0^2 + 4L_\mathrm{s} \omega_0^2 + 4R_2 T_\mathrm{s} \omega_0^2 + 2T_\mathrm{s} K_\mathrm{p} \omega_0^2 \\
&a_7 = 4R_2 \omega_0^2 - 4K_\mathrm{p} \omega_0^2
\end{aligned}
$$

The self-adaptivity requires that the PR controller can react to different operating frequencies with the optimal response[10]. Therefore, the spectrum characteristics of the controller, determined by K_p and K_r, ought to vary with different operating frequencies. In our design, we set different frequency-domain characteristics by altering K_p and K_r in different frequency ranges, thus ensuring the optimal performance of the controller.

According to **Supplementary** Eqs. (14)-(15) and the parameters in **Supplementary** Table 2, the root locus can be plotted as shown in **Supplementary** Figs. 2**d** and **e**. The poles remained at the left half of the complex plane under a wide range of control parameters. For instance, when $K_\mathrm{r} < 7250$, the system's poles are all still located on the left half of the complex plane. When K_r further increases, the system poles will move to the right half plane, which means that the overall system tends to be unstable[11].

In addition, only when f_s is determined, as a linearized time-invariant system, the dynamic performance and stability of the proposed non-Foster-inspired circuit can be analyzed by the open-loop gain/phase margins and closed-loop bandwidth as shown in **Supplementary** Figs. 2**f** and **g**. Consequently, the parametric design of PR controller for stability is easy to achieve.

Supplementary Note 3: The program flowchart of software design for the digital non-Foster-inspired electronics. The software design primarily consists of main program and interrupt service routines (ISRs).

The DSP (TMS320F28335, Texas Instruments) is used to execute programs here. In the main program (**Supplementary** Fig. 3**a**), initialization of peripherals and PR controller, as well as the relevant interrupt configuration is complete. Afterwards, the DSP remains in a waiting state until interrupts occur.

The purpose of the timer interrupt is to generate the PWM output pulses, and the period of the pulse is equal to that of the timer. Whenever the counter overflows, an interrupt will be generated by the timer. Then, an interrupt request will be sent to the CPU where the ISR is handled. Meanwhile, the program pointer will be automatically located to the starting address of the ISR function[12]. The primary work of the timer ISR, shown in **Supplementary** Fig. 3**b**, is divided into the following steps

(1) The DSP generates triggers for starting A/D (AD7656, Analog Devices) conversion to collect and restore the actual electrical signals (u_s and i_o) at the moment.

(2) Generation of the reference signal i_{ref}. The reference current i_{ref} is generated according to u_s and the impedance reference $Z_{ref} = R_{ref} + jX_{ref}$. i_{ref} needs to be discussed respectively in relation to the magnitude of the reactance reference X_{ref} and the transducer total reactance X_T. Under those two cases, i_{ref} can be expressed as

$$\begin{cases} -\dfrac{1}{\omega_s\left(X_T+X_{ref}\right)}\dfrac{\mathrm{d}\left[u_s-i_{ref}\left(R_T+R_{ref}\right)\right]}{\mathrm{d}t}=i_{ref} & X_T+X_{ref}>0 \\ \dfrac{\left(X_T+X_{ref}\right)}{\omega_s}\dfrac{\mathrm{d}i_{ref}}{\mathrm{d}t}=u_s-i_{ref}\left(R_T+R_{ref}\right) & X_T+X_{ref}<0 \end{cases} \tag{16}$$

After discretization with ZOH [13], the relation between i_{ref} and u_s can be expressed as[14]

$$\begin{cases} i_{ref}(k)=\mathrm{A}i_{ref}(k-1)+\mathrm{B}u_s(k-1) & X_T>X_{ref} \\ i_{ref}(k)=\mathrm{C}i_{ref}(k-1)+\mathrm{D}\left(u_s(k)-u_s(k-1)\right) & X_T<X_{ref} \end{cases}, \tag{17}$$

where coefficients A, B, C and D are respectively

$$\begin{cases} \mathrm{A}=e^{-\left(\left(R_T+R_D\right)\omega_s T_s\right)/\left(X_T+X_D\right)} \\ \mathrm{B}=\left(1-A\right)/\left(R_T+R_D\right) \\ \mathrm{C}=e^{\left(\left(X_T+X_D\right)\omega_s T_s\right)/\left(R_T+R_D\right)} \\ \mathrm{D}=1/\left(R_T+R_D\right) \end{cases}, \tag{18}$$

R_{T} and X_{T} are the resistance and reactance of the electroacoustic transducer, respectively. R_{D} and X_{D} manifest the equivalent negative R and C/L of the digital non-Foster-inspired circuit.

(3) Subsequently, the error between i_{ref} and the output current i_{o} will be calculated, which is the input of the PR controller.

(4) Tustin transform[15] **Supplementary** Eq. (19) is employed to discretize the PR controller

$$\begin{cases} G_{\mathrm{PR}}(s) = K_{\mathrm{p}} + K_{\mathrm{r}} \dfrac{2\omega_{\mathrm{c}} s}{s^2 + 2\omega_{\mathrm{c}} s + \omega_0^2} \\ s = \dfrac{2}{T_{\mathrm{s}}} \dfrac{1-z^{-1}}{1+z^{-1}} \end{cases}. \tag{19}$$

By solving it, the PR controller can be discretized as

$$G_{\mathrm{PR}}(z) = \frac{K_{\mathrm{p}} \left[\dfrac{2}{T_{\mathrm{s}}} \dfrac{1-z^{-1}}{1+z^{-1}} \right]^2 + 2\omega_{\mathrm{c}} \left(K_{\mathrm{p}} + K_{\mathrm{r}} \right) \dfrac{2}{T_{\mathrm{s}}} \dfrac{1-z^{-1}}{1+z^{-1}} + K_{\mathrm{p}} \omega_0^2}{\left[\dfrac{2}{T_{\mathrm{s}}} \dfrac{1-z^{-1}}{1+z^{-1}} \right]^2 + 2\omega_{\mathrm{c}} \dfrac{2}{T_{\mathrm{s}}} \dfrac{1-z^{-1}}{1+z^{-1}} + \omega_0^2}. \tag{20}$$

Eventually, it can be can be further simplified as

$$G_{\mathrm{PR}}(z) = \frac{\mathrm{A}_2 z^2 + \mathrm{A}_1 z + \mathrm{A}_0 z^0}{\mathrm{B}_2 z^2 + \mathrm{B}_1 z + \mathrm{B}_0 z^0}, \tag{21}$$

where the coefficients A_i and B_i (i=0, 1, 2) are

$$\begin{cases} \mathrm{A}_2 = 4K_{\mathrm{p}} + 4\omega_{\mathrm{c}} T_{\mathrm{s}} \left(K_{\mathrm{p}} + K_{\mathrm{r}} \right) + K_{\mathrm{p}} \left(\omega_0 T_{\mathrm{s}} \right)^2 \\ \mathrm{A}_1 = -8K_{\mathrm{p}} + 2K_{\mathrm{p}} \left(\omega_0 T_{\mathrm{s}} \right)^2 \\ \mathrm{A}_0 = K_{\mathrm{p}} \left(\omega_0 T_{\mathrm{s}} \right)^2 - 4\omega_{\mathrm{c}} T_{\mathrm{s}} \left(K_{\mathrm{p}} + K_{\mathrm{r}} \right) + 4K_{\mathrm{p}} \\ \mathrm{B}_2 = 4 + 4\omega_{\mathrm{c}} T_{\mathrm{s}} + \left(\omega_0 T_{\mathrm{s}} \right)^2 \\ \mathrm{B}_1 = -8 + 2\left(\omega_0 T_{\mathrm{s}} \right)^2 \\ \mathrm{B}_0 = \left(\omega_0 T_{\mathrm{s}} \right)^2 - 4\omega_{\mathrm{c}} T_{\mathrm{s}} + 4 \end{cases}. \tag{22}$$

(5) Closed-loop feedback regulation. After comparing the output of PR controller with the triangular carrier wave[16], the pulse sequences outputted from the ePWM module will be adjusted to the action of switching devices towards the direction of the error reduction[17]. Since the timer ISR will be responded whenever the counter overflows, the PR controller can continuously adjust the error to realize the zero steady-state error of the sinusoidal reference current signal by a large gain over a

wide bandwidth and the output characteristics of the designed digital non-Foster-inspired circuit will also eventually satisfy the requirements.

As soon as the DSP detects four edges (rising edges or falling edges), CPU will handle the eCAP ISR shown in **Supplementary** Fig. 3**c**[12]. The primary work of the eCAP ISR is divided into the following steps.

(1) Frequency tracking. The operation frequency f_s is calculated according to the intervals between the recorded edges, thereby achieving the frequency tracking. Because the eCAP module of DSP 28335 requires to capture at least one rising edge and one falling edge, the acquisition time for accurate frequency estimation below 2 kHz takes no less than half a cycle.

(2) Set the impedance reference Z_{ref}. Z_{ref} should be set according to the operation frequency f_s so that the expected terminal negative R and L/C of the digital non-Foster-inspired circuit presents any engineered frequency dispersion. By mean of the digital feedback control, the designed circuit can ultimately transform Z_{ref} to $Z_D = -Z_{\text{ref}}$.

(3) Self-adaptive adjustment. According to f_s, the program selects corresponding PR controller parameters and pre-set target parameters, which ensures that the PR controller can respond to the system operating status with optimal control characteristics. It is, therefore, self-adaptive. Here, see **Supplementary** Table 1 for detailed parameter configurations.

Supplementary Note 4: Conversion of vibration velocity to sound pressure. With a rigid flat circular piston mounted in an infinite flat baffle, the peak radiated sound pressure P can be derived according to **Supplementary** Eq. (23), when the measurement distance h varies from zero to infinity along the piston axis[18]

$$P = \iint \mathrm{d}p = \iint j\frac{k\rho_0 c_0}{2\pi h} v_{\mathrm{D}} \mathrm{e}^{-jkh}\mathrm{d}S\,, \tag{23}$$

where $k = \omega/c_0$ represents the wave number.

First, when r varies from zero to r_1 as shown in **Supplementary** Fig. 4, P_1 can be solved by

$$P_1 = \iint j \frac{k\rho_0 c_0}{2\pi h_1} v_{\mathrm{D}} e^{-jkh_1} dS_1 , \tag{24}$$

Supplementary Eq. (25) illustrates the calculation method of the infinitesimal $\mathrm{d}S_1$

$$\begin{cases} dS_1 = 2\pi r \cdot l_1 d\theta \\ \theta = \arcsin \dfrac{r}{l_1} \\ d\theta = \dfrac{1}{\sqrt{l_1^2 - r^2}} dr \\ h_1 = \sqrt{l_1^2 + \left(\sqrt{l_1^2 - r_1^2} + z\right)^2 - 2 \cdot l_1 \cdot \left(\sqrt{l_1^2 - r_1^2} + z\right) \cos\theta} \end{cases} . \tag{25}$$

Next, when *r* varies r_1 from to r_2 as shown in **Supplementary** Fig. 4, P_2 can be solved as well by

$$P_2 = \iint j \frac{k\rho_0 c_0}{2\pi h_2} v_{\mathrm{D}} e^{-jkh_2} dS_2 . \tag{26}$$

Similarly, **Supplementary** Eq. (27) illustrates the calculation method of the infinitesimal $\mathrm{d}S_2$

$$\begin{cases} dS_2 = \dfrac{1}{\sin\varphi} \cdot 2\pi r dr \\ h_2 = \sqrt{\left(\dfrac{r}{\sin\varphi}\right)^2 + \left(\sqrt{l_2^2 - r_1^2} + z\right)^2 - 2 \cdot \dfrac{r}{\sin\varphi} \cdot \left(\sqrt{l_2^2 - r_1^2} + z\right) \cos\varphi} \end{cases} . \tag{27}$$

Finally, the SPL in front of the transducer at the axial position (*z*, 0) can be obtained by **Supplementary** Eq. (28). Here, the reference sound pressure P_{ref} is 2×10^{-5} Pa[19]

$$\mathrm{SPL} = 20\log_{10}\left(P_1 + P_2\right) - 20\log_{10}\left(p_{\mathrm{ref}}\right) . \tag{28}$$

Supplementary Note 5: Limitations discussion and error analysis. There are some necessary instructions that need to be completed for interrupt handling, including interrupt request, interrupt response, returning from interrupt and so on[12]. To ensure complete and accurate operation of each ISR, the sampling time of DSP cannot be less than 25 μs, i.e., the switching frequency should be below 40 kHz.

The PWM technique is used to build up a sinusoidal waveform with multiple pulses whose duty cycle vary sinusoidally with time. Its rational is to achieve low harmonics[6]. Furthermore, the cutoff frequency of the LC filter used in the proposed digital non-Foster-inspired circuit is cautiously selected to be much less

than the switching frequency f_{sw} so as to filter the higher harmonics out[20]. In this work, when f_{sw}=40kHz, the cutoff frequency of the LC filter is suggested to be below 4 kHz.

In addition, it is apparent that the proposed self-adaptive PR controller must have an infinite gain at the operating frequency f_s to realize the desired negative impedance tracking with zero sinusoidal steady-state error when f_s is equal to the resonance frequency of PR controller f_0.

The proposed adaptive PR controller was discretized by Tustin method as **Supplementary** Eq. (19), which leads a deviation on Δf_0 between f_0 (equal to f_s) , the resonance frequency of PR controller in *s*-domain, and f_{d0}, the resonance frequency of PR controller in *z*-domain. It can be seen from **Supplementary** Fig. 5**a** that Δf_0 with respect to the expected f_s becomes greater as the sampling time ($1/f_{sw}$) and f_s increase. At an operating frequency of 1400 Hz, Δf_0 will reach 20 Hz at f_{sw}=40 kHz. It means a significant gain loss as shown in **Supplementary** Fig. 5**b**. Although the controller gain can be improved by parameter adjustment, the adjustment process is also limited by stability and transient performance. When f_s is far beyond the resonant point ($f_s > 100\text{Hz}$), the steady-state errors as a whole rise progressively due to the gain loss. When f_s is equal to 1400 Hz, the error of the negative resistance is up to 13.72% while that of the negative inductance is 11.81%.

In order to quantify the control precision, the equivalent negative R and L/C with or without FD are measured through the power analyzer. The errors between the expected values and actual values have been displayed. According to **Supplementary** Figs. 5**b** and **c**, the closer the frequency is to the resonant point (40~100 Hz), the larger the steady-state errors between the desired and obtained equivalent impedance. This is because there is appreciable distortion in the actual voltage and current waveforms near the resonant frequency of transducer[21]. This causes the imprecise measurement of the transducer impedance and the inaccurate sensing of i_{ref} for the controller.

Supplementary Note 6: Power and loss calculation of the digital non-Foster-inspired electronics. The

usage of switch-mode electronics breaks the power limitation of conventional analog op-amp-based non-Foster circuits. The cancellation of excess stored energy through the digital non-Foster-inspired electronics makes sure that the negative reactance $L_D(\omega)$ is equal to the eddy current reactance of the high power transducer $L_E(\omega)$. Simultaneously, the negative resistance $R_E(\omega)$ of the digital non-Foster-inspired electronics ensures more energy radiation from the transducer over a wide bandwidth. With the equivalent negative R and L with FD ($Z_D(\omega) = R_D(\omega) + j\omega L_D(\omega)$) and the output current i_o (its root mean square is I_o), the apparent power S_D can be derived as

$$S_D = I_o^2 \sqrt{\left(R_D(\omega)\right)^2 + \left(\omega L_D(\omega)\right)^2}\,. \tag{29}$$

According to the measurement results UDF_5 , UDF_3 and i_o in Fig. 4**b** of the main text, when f_s is 600 Hz or 1200 Hz, S_D is 107.54 VA and 229.99 VA, respectively. For the conventional analog non-Foster circuit[4], the maximum apparent power is approximately 0.44 VA based on the manifested impedance and the op-amp supply voltage. By contrast, the power level of the digital non-Foster circuit is upgraded by about three orders of magnitude.

However， the power loss generated by switching devices can be further divided into on-state loss, off-state loss and switching loss[6]. In general, the off-state leakage current is very small so that the off-state loss is negligible. For the switching devices S_1~S_4 used in our designed circuit, S_1 and S_2 operate at $f_{sw} = 40\text{kHz}$ while S_3 and S_4 are operated at f_s . Consequently, the losses of S_1 and S_2 are mainly the switching losses while those of S_3 and S_4 are the on-state losses.

The switching energy E_{sw} consists of turn-on switching energy E_{on} and turn-off switching energy E_{off} , which can be solved through the integral of product of drain-source voltage $V_{ds}(t)$ and the drain current $I_d(t)$ during the switching transient. Therefore, E_{sw} , E_{on} and E_{off} can be defined

$$\begin{cases} E_{\mathrm{sw}} = E_{\mathrm{on}} + E_{\mathrm{off}} \\ E_{\mathrm{on}} = \int_0^{T_{\mathrm{on}}} V_{\mathrm{ds}}(t) I_{\mathrm{d}}(t)\,\mathrm{d}t \\ E_{\mathrm{off}} = \int_0^{T_{\mathrm{off}}} V_{\mathrm{ds}}(t) I_{\mathrm{d}}(t)\,\mathrm{d}t \end{cases}. \tag{30}$$

T_{on} and T_{off} have been defined as switching-on and switching-off duration, respectively[22]. According to **Supplementary** Eq. (30), the double-pulse simulation test[23] is used to estimate E_{sw} under the high-power scenario shown in Figs. 5**b** and **c** of the main text. Here, V_{ds} is set as 300 V, identical with the experimental setting V_{DC}. Since i_{o} is sinusoidal with a RMS value of approximate 2 A, I_{d} at 3 A is selected to estimate P_{sw}. A RC snubber is added to suppress fully switching oscillation[24]. Eventually, the switching waveforms of both turn-on and turn-off transients are shown in the **Supplementary** Figs. 6**a** and **b**. Also, considering that both S_1 and S_2 operate at f_{sw}, P_{sw} can be solved.

$$P_{\mathrm{sw}} = 2 f_{\mathrm{s}} E_{\mathrm{sw}}. \tag{31}$$

E_{sw} is about 21.12 μJ. Thus, P_{sw} is estimated to be 1.7 W.

P_{on} can be obtained via the drain-source on-state resistance $R_{\mathrm{ds(on)}}$ provided by the datasheet of SiC MOSFET[25] (C3M0060065D, Cree). Additionally, both S_3 and S_4 have the on-state losses, and each device is only conducting state for half operating cycle. Therefore, P_{on} can be solved

$$P_{\mathrm{on}} = I_{\mathrm{d}}^2 R_{\mathrm{ds(on)}}, \tag{32}$$

P_{on} is 0.54 W . The above analysis confirms that the power loss generated by switching devices occupied a very small part of its apparent power.

Supplementary Note 7: Analysis and optimization for transient response time. The transient process by our approach consists of three main steps: estimation of f_{s}, adjustment of PR control parameters, and transient response of the system. Among them, the parameter calculation and adjustment process can be optimized by increasing the processing speed of DSP. Moreover, the transient response to steady-state can also be optimized by the adjustment of the PR parameters. Finally, the process of operating frequency estimation can be saved by algorithm optimization in practical applications.

One of the feasible methods is delayed synchronous operation. When the first command code is received, the frequency estimation and parameter calculation are carried out first, and then, the DSP waits for the arrival of the next command code. When the second command code is received, the modulated signal of the previous command code is transmitted synchronously to achieve negative impedance matching with the proposed circuit. At this moment, owing to the accurately known frequency and corresponding controller parameters, the transient process only consists of the dynamic response.

We have experimentally tested this idea. In this way, the proposed circuit can ensure the synchronous operation with the signal source, which greatly reduces the transient response process of the proposed digital non-Foster-inspired electronics, as shown in **Supplementary** Fig. 7.

Supplementary Note 8: The experimental design of image transmission over airborne acoustic channels. The image transmission experiments over airborne acoustic channels are carried out with the frequency shift keying (FSK) technique[26]. The receiver node is installed to an open playground at a distance of approximately 60 meters from the transmission node, which minimized the impact of acoustic reflection and environmental noises on reception, as illustrated in Fig. 4**c** of the main text.

The operation of the complete experiment is introduced in detail as follows:

(1) The first step is to select a picture. The transmitted image of the Hunan University in Chinese characters with a specific spatial resolution of 62×175 pixels coded at 8 bits is converted from the original image.

(2) A digital signal is generated by using FSK modulation as illustrated in **Supplementary** Fig. 8. The modulation process divides the transmitted image into pixels, with each individual pixel being a different color[27]. The color value of each pixel is then sequentially transmitted starting from the top left of the image. Next, the process is repeated for the next line of pixels until all lines of pixels have been sent. We carefully selected the FSK frequency to distinguish the bandwidth of the electroacoustic transducer, where yellow, red,

blue, black, and white are represented by 200 Hz tone, 300 Hz tone, 600 Hz tone, 1000 Hz tone, and 1200 Hz tone, respectively. The duration of ten cycles is used for each tone. Each line is transmitted followed by 500 Hz tone of sixteen cycles as the line break signal[28].

(3) After converted to analog audio by the soundcard of a PC, FSK modulated waveforms are fed into the transducer loaded by different impedance matching approaches.

(4) To transmit information, the receiving process occurs simultaneously on the receiver node. FSK transmission waveforms are first picked up by the acoustic sensor (ISV1610, Hangzhou Aihua Instruments Co., Ltd). According to **Supplementary** Fig. 9**a**, the FSK demodulation process is divided into the following five parts.

1) We have to translate analog FSK tones into digital data by a soundcard with a sampling frequency f_a of 44.1 kHz at 16 bits per sample.
2) Digital processing of the demodulated signal can significantly improve the picture quality especially when the environment is noisy[28]. Inspired by slow-scan TV transmission, we adopt an approach as shown in **Supplementary** Fig. 9**b** to process signals for restoring image quality. The received digital FSK tones y(k) goes through the finite impulse response filters[29] to obtain each modulation frequency separately, and then consistency of each modulation frequency is guaranteed by normalization. Finally, all the modulated signals are added together to generate the processed audio signal yy(k).
3) By picking up the signal peaks, the locations of the line-break frequency are retrieved. Therefore, we decouple the demodulation process of each line to avoid mutual influence.
4) Discrete Fourier Transformation (DFT) is used to identify the modulation frequency of each pixel signal[30]. The pointer m points to the line being demodulated and the pointer k points to the position of the pixel being identified. B represents the signal length for DFT, which is equal to 222 here to ensure that all six modulation frequencies can be accurately identified. To uniformly compare the image

receiving effect under different impedance matching approaches, the threshold values (TH) of the same color are set to be identical. The THs of yellow, red, blue, and black are respectively 0.02, 0.07, 0.19, 0.1. If the actual value after processing is greater than TH, the pixel is received; otherwise, it is shown in white.

5) Recover color information from the FSK-modulated signals and generate the received image.

Eventually, an exclusive program using the described algorithms is designed and tested. The resultant images are shown in Fig. 5**c** of the main text.

Supplementary Figures

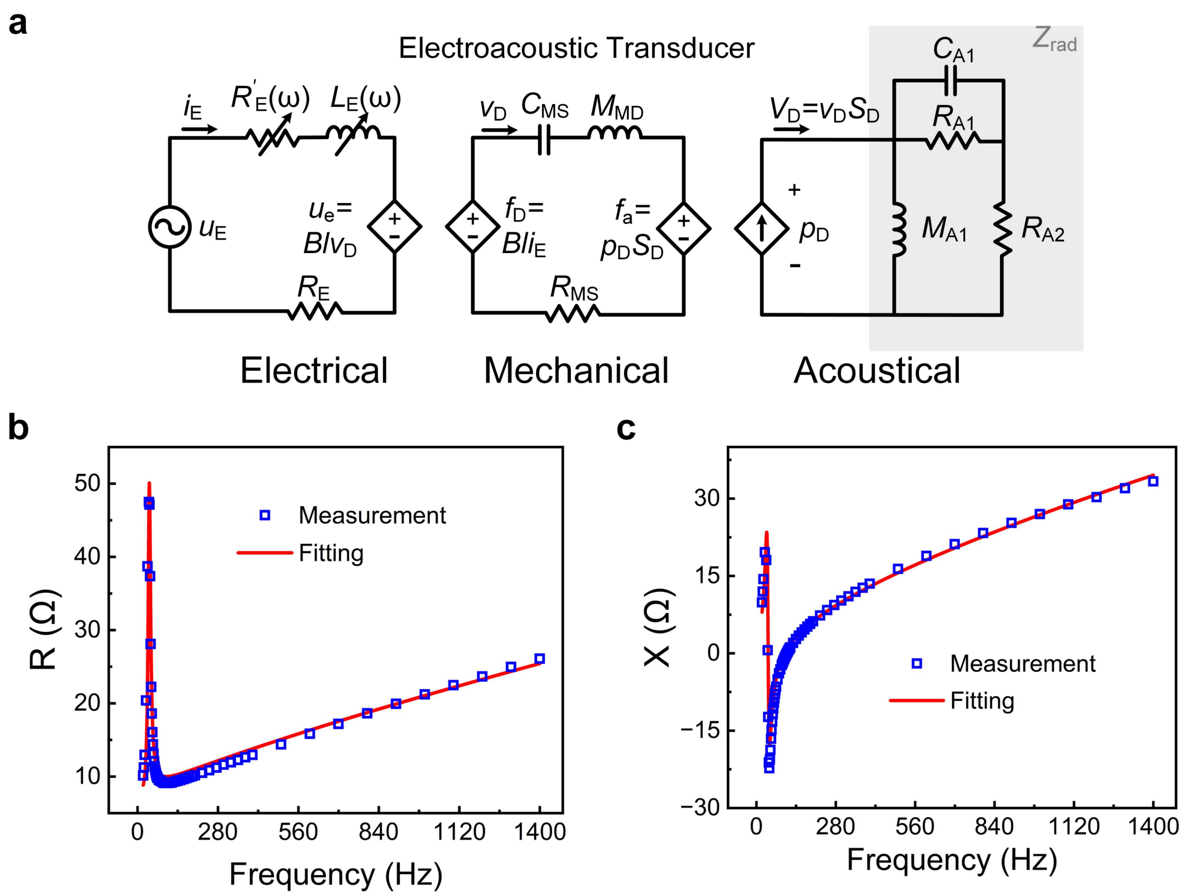

Supplementary Figure 1 | The impedance model of the electroacoustic transducer. a. The analogous circuit for the electroacoustic transducer in an infinite baffle. b, c. Comparison of measured and fitted impedances of the electroacoustic transducer. The resistive component and reactance component are plotted respectively in b and c. The blue dots indicate the measurement result and the red lines indicate the fitting result.

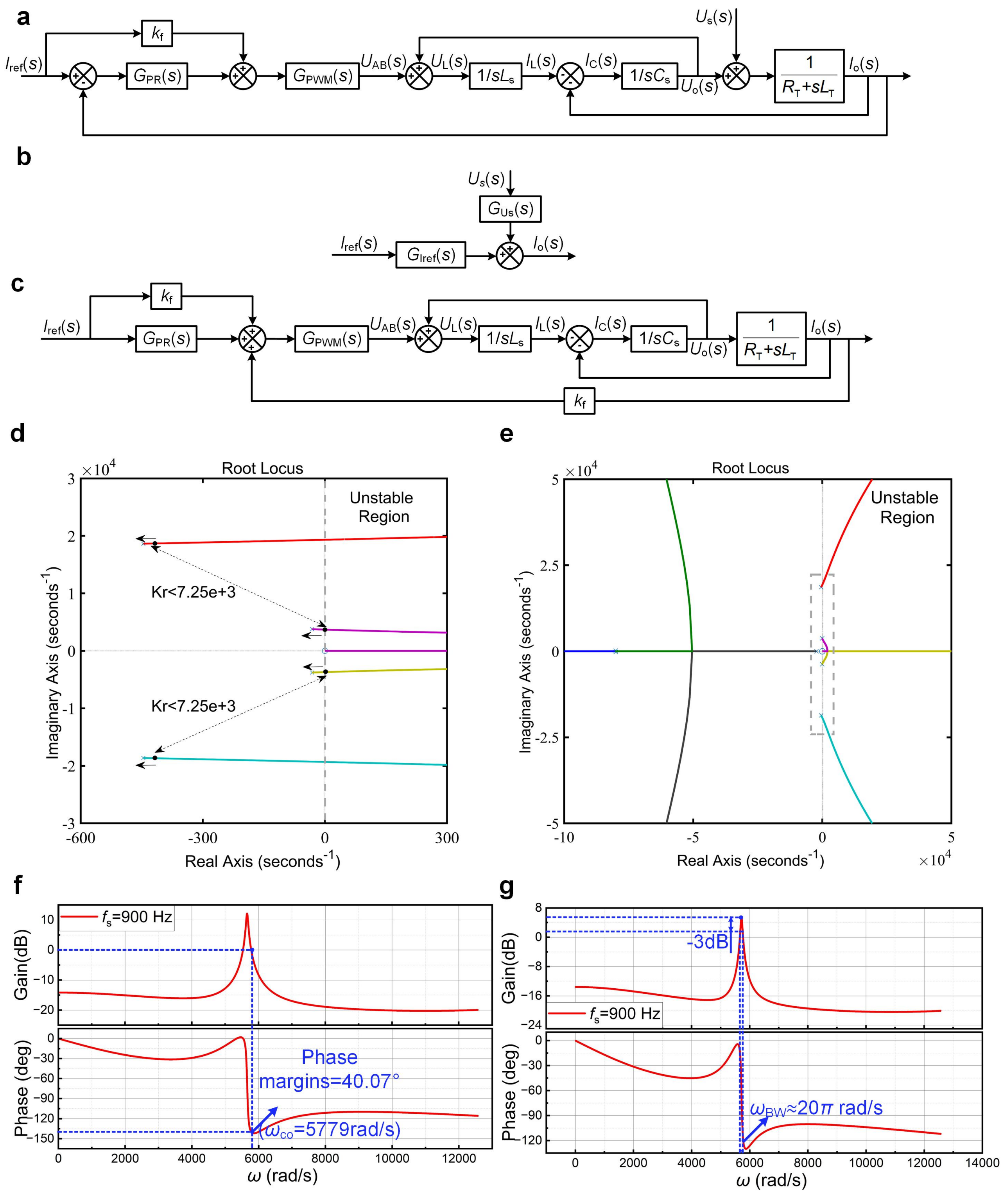

Supplementary Figure 2 | Stability analysis of the digital non-Foster-inspired electronics. a. The closed-loop transfer function block diagram of the digital non-Foster-inspired electronics. b. The simplification result for the closed-loop transfer function block diagram. Here, $G_{\mathrm{ref}}(s)$ is the transfer function from the input node $I_{\mathrm{ref}}(s)$ to the output node $I_{\mathrm{o}}(s)$ when $U_{\mathrm{s}}(s)=0$. $G_{\mathrm{Us}}(s)$ is the transfer function from the input node $U_{\mathrm{s}}(s)$ to the output node $I_{\mathrm{o}}(s)$ when $I_{\mathrm{ref}}(s)=0$. c. The open-loop transfer

function block diagram of the digital non-Foster-inspired electronics. d. Root locus diagram of $D(\mathrm{s})=0$ with the parameter K_{r} . e. Enlarged root locus diagram of (g). f. The open-loop bode plot of the digital non-Foster-inspired electronics at an operating frequency f_{s} of 900 Hz. ω_{co} indicates the cutoff frequency at which the open-loop gain is 0 dB. g. The closed-loop bode plot of the digital non-Foster-inspired electronics at an operating frequency f_{s} of 900 Hz. ω_{BW} indicates the closed-loop bandwidth, which is the frequency range of -3 dB down from the peak gain.

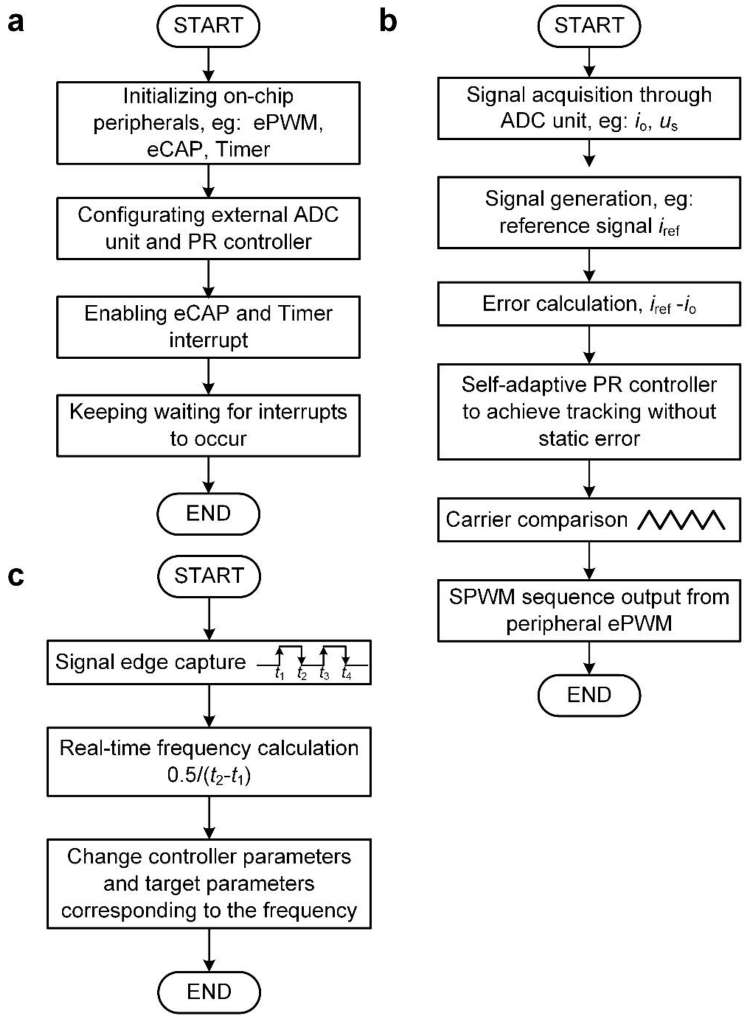

Supplementary Figure 3 | The software program flowchart of the digital non-Foster-inspired circuit. a. The flowchart for the main program with initialization. b. The flowchart for the timer ISR. c. The flowchart for the eCAP ISR.

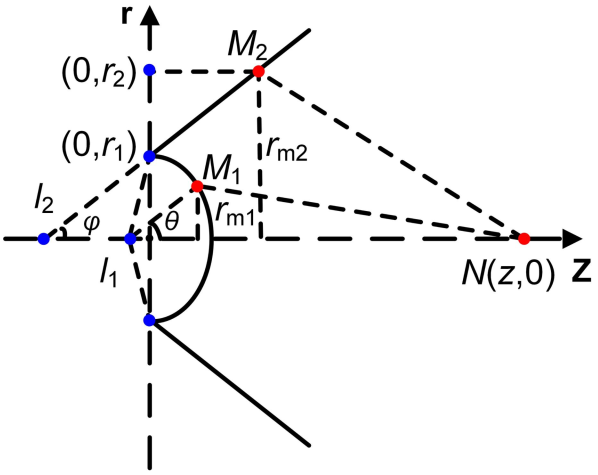

Supplementary Figure 4 | Schematic diagram for the solution of sound pressure level (SPL) when *r* varies from zero, r_1 to r_2. According to the calculation method of radiated sound pressure, the equation of SPL can be derived. M_1 represents the infinitesimal face when *r* varies from zero to r_1. M_2 represents the infinitesimal face when *r* varies r_1 from to r_2. $N(z,0)$ represents the measurement point on the central axis of the transducer.

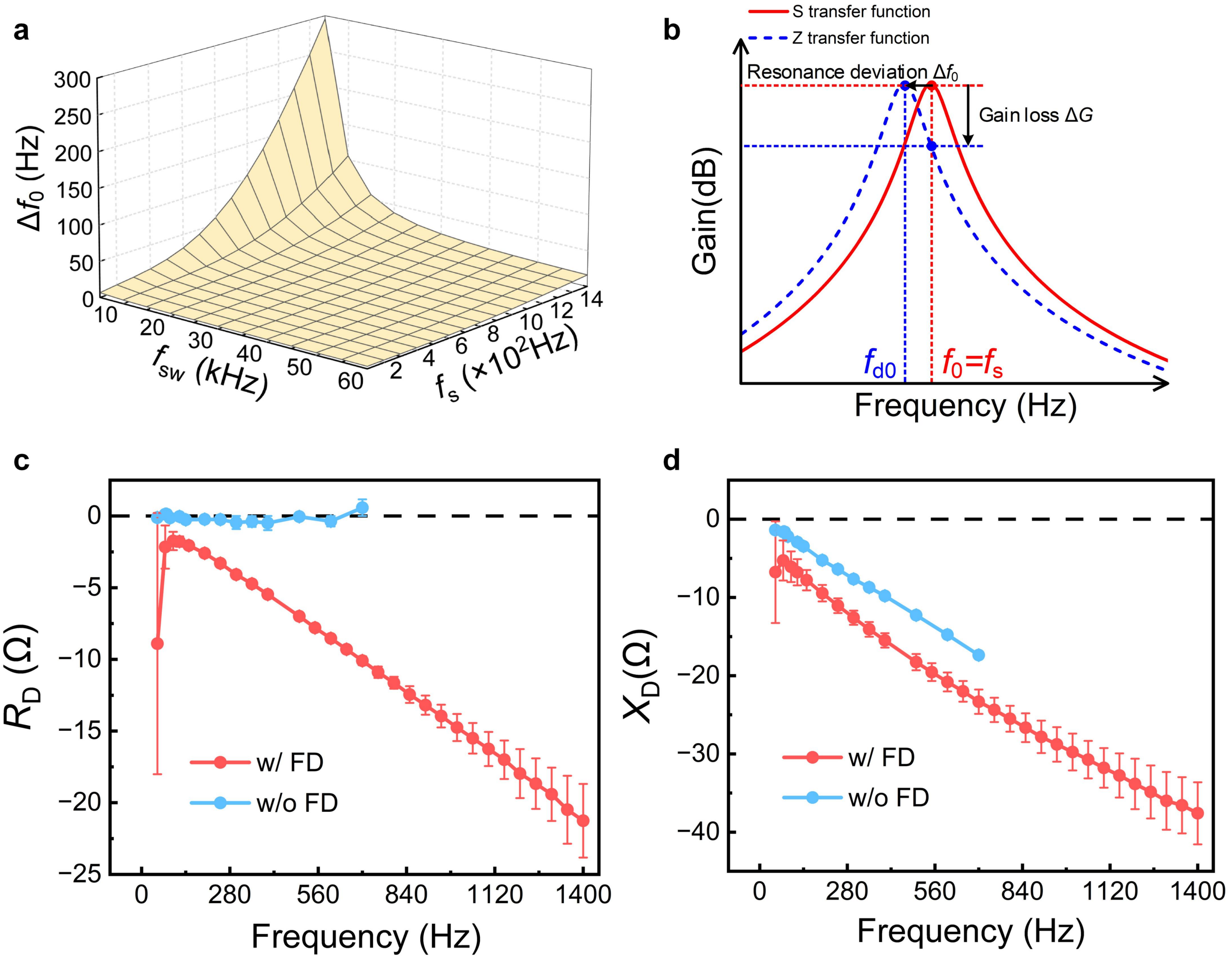

Supplementary Figure 5 | The diagram of the actual control error. (a) The frequency deviation Δf_0 between the resonance frequency of PR controller in *s*-domain f_0 (equal to f_s) to the resonance frequency of PR controller in *z*-domain f_{d0}. f_{sw} is the sampling frequency that is equal to the switching frequency of SiC MOSFETs in the proposed circuit. (b) Schematic diagram of gain loss caused by frequency deviation of PR controller discretization. (c) and (d) The measured negative resistive component (c) and negative reactance component (d) with (red lines) or without (blue lines) FD are plotted respectively. The error bars represent the numerical errors between the expected values and actual values.

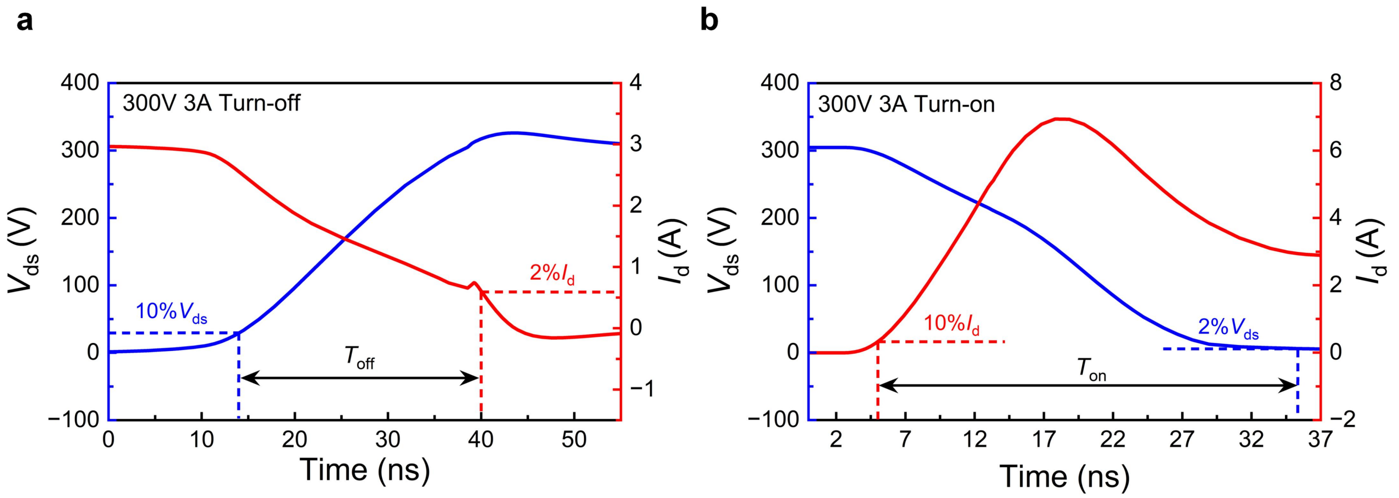

Supplementary Figure 6 | Switching transient processes for loss estimation. a. Turn-on waveform b. Turn-off waveform.

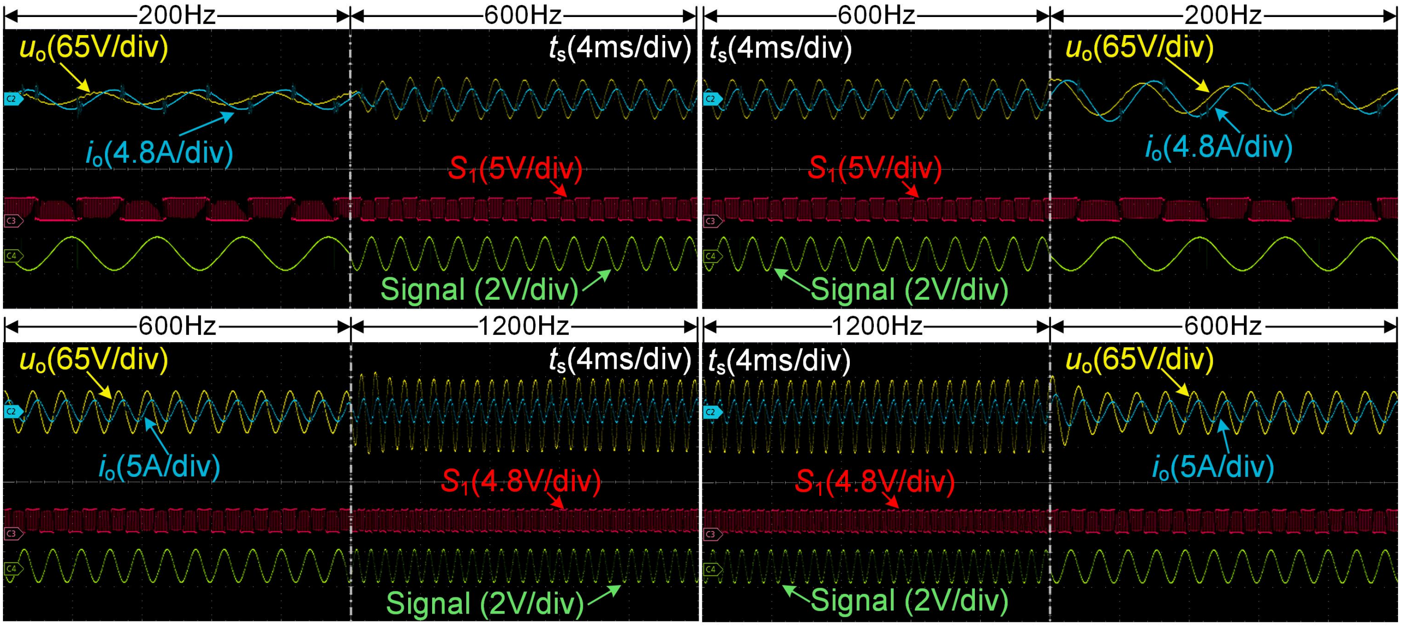

Supplementary Figure 7 | Transient response of the digital non-Foster-inspired circuit after algorithm optimization. The yellow curve indicates terminal voltage u_o of the digital non-Foster-inspired circuit, while the blue one is its output current i_o. S_1 is the control signal of the SiC MOSFET represented by the red curve and the green curve indicates the waveform generated by the signal generator.

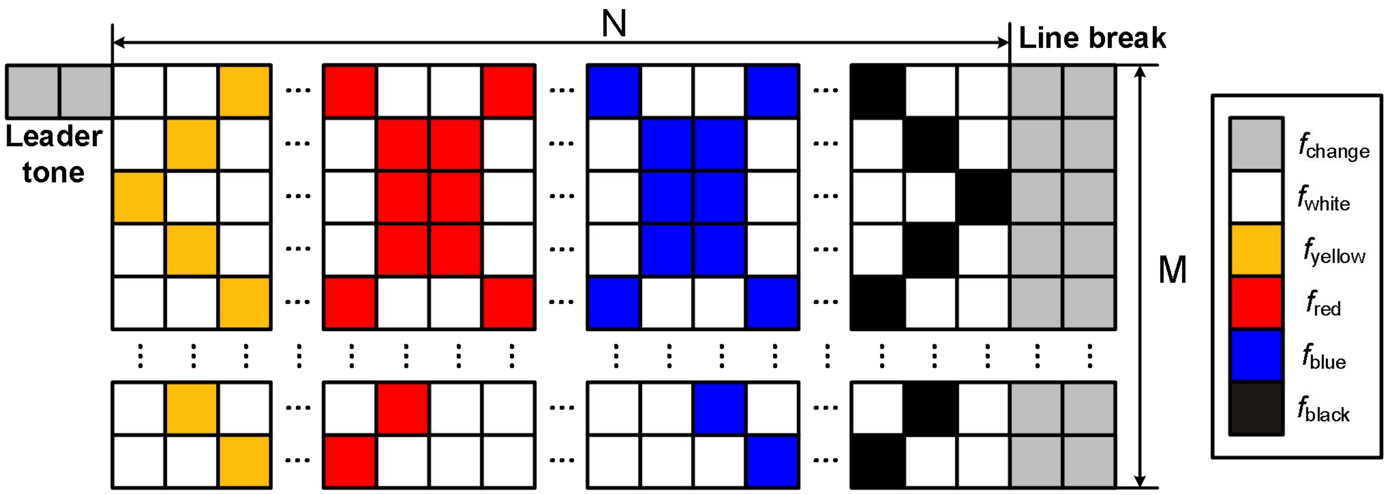

Supplementary Figure 8 | Schematic diagram for modulation mode. The format of the transmitted image is M lines and N pixels per line, with each pixel represented by a modulation frequency, including five colors: white, blue, black, red, and yellow. f_{change} represents the frequency of the leader tone signal and the line break signal.

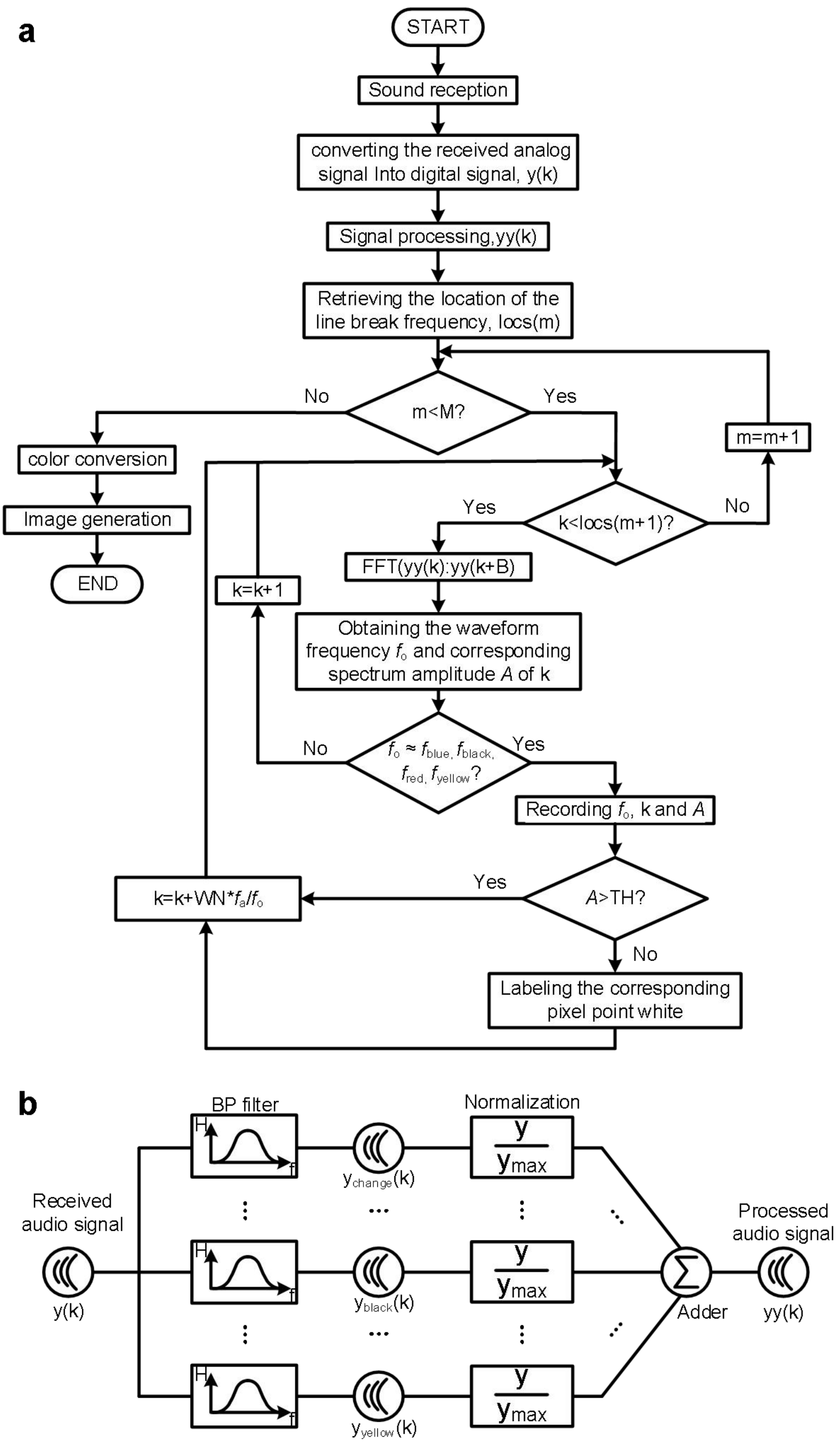

Supplementary Figure 9 | The flowchart of the image demodulation process. **a.** The demodulation converts the acoustic signal picked up by acoustic sensor into an output image. b. Digital signal processing for the received audio.

Supplementary Tables

Supplementary Table 1 | Parameters adjustment with the operation frequency f_s . The values of PR controller parameters (K_p , K_r) and feedforward gain (K_f) need to satisfy not only the control requirements but also the stability of the system. ω_c is the cutoff bandwidth. R_T and X_T represent the resistance and reactance of electroacoustic transducer, respectively. R_D and X_D represent the manifested negative resistance and inductance with FD of the proposed digital non-Foster-inspired circuit, respectively.

Operating frequency f_s	K_f	K_p	K_r	ω_c	$R_T + R_D$	$X_T + X_D$
40 Hz	3	2	100	10π	48.64784	8.50134
100 Hz	3	0.7	40	10π	7.99155	−5.00865
200 Hz	3	0.7	40	10π	7.51278	−2.19929
300 Hz	3	2	60	10π	7.44784	−1.43348
400 Hz	3	1	60	10π	7.42648	−1.06676
500 Hz	3	1	60	10π	7.41682	−0.85035
600 Hz	3	1	60	10π	7.41164	−0.70725
700 Hz	3	3	60	10π	7.40853	−0.6055
800 Hz	3	1.5	60	10π	7.40652	−0.52941
900 Hz	3	1.5	150	10π	7.40515	−0.47034
1000 Hz	3	2	100	10π	7.40416	−0.42315
1100 Hz	3	3	150	10π	7.40344	−0.38458
1200 Hz	3	3	200	10π	7.40289	−0.35246
1300 Hz	3	5	250	10π	7.40246	−0.32529

Supplementary Table 2 | The system parameters. The parameters are used to solve the root locus.

Main circuit parameters	Control parameters
$L_s = 2\text{mH}$; $C_s = 2\mu\text{F}$; $R_T = 15.83\Omega$;	$\omega_c = 10\pi$; $\omega_0 = 1200\pi$; $K_f = 1$; $K_p = 3$;
$L_T = 5.01\text{mH}$;	$T_s = 2.5\times10^{-5}\text{s}$;

Supplementary Table 3 | The part number for all the main components.

Component	Model	Producer
AC capacitor C_{AC}	350V 10uF film capacitor	TDK Electronics AG, Germany
Current sensor	LA35-NP	LEM Electronics, Switzerland
Voltage sensor	TV16E	Dechang Electric Co., Ltd., China
DC/DC module	VRA2415ZP-10WR3	MORNSUN Technology Co., Ltd., China
	IB2403LS-1WR3	MORNSUN Technology Co., Ltd., China
ADC module	AD7656	Analog Devices, Inc., America
DSP	TMS320F28335	Texas Instruments, Inc., America
Module driver	UCC21520	Texas Instruments, Inc., America
SiC MOSFET	C3M0060065D	Wolfspeed, Inc., America
DC-link capacitor C_{DC}	450V 820uF electrolytic capacitor	Nippon Chemi-Con Corp., Japan
	350V 10uF film capacitor	TDK Electronics AG, Germany
Electroacoustic transducer	DL100LLB-01	Dongguan Huachuang Audio Equipment Co., Ltd, China

Supplementary References

1. Mojahed, A., Bergman, L. A. & Vakakis, A. F. Generalization of the concept of bandwidth. *J. Sound Vib.* **533**, 117010 (2022).

2. Leach, W. M. Introduction to electroacoustics and audio amplifier design 85-105 (Kendall Hunt, Dubuque, IA, 2010).

3. Wright, J. R. An empirical model for loudspeaker motor impedance. *J. Audio Eng. Soc.* **38**, 749-754 (1990).

4. Harris, N. J. & Hawksford, M. O. J. Introduction to distributed mode loudspeakers (DML) with first-order behavioural modelling. *IEE Proc.-Circuits Devices Syst.* **147**, 153-157 (2000).

5. Vidal, A. *et al.* Assessment and optimization of the transient response of proportional-resonant current controllers for distributed power generation systems. *IEEE Trans. on Ind. Electron*. **60**, 1367-1383 (2012).

6. Erickson, R. W. & Maksimovic, D. Fundamentals of power electronics (Springer, New York, NY, 2007).

7. Li, Y. W., Loh, P. C., Blaabjerg, F. & Vilathgamuwa, D. M. Investigation and improvement of transient response of DVR at medium voltage level. *IEEE Trans. Ind. Appl.* **43**, 1309-1319 (2007).

8. Li, H. *et al.* A time-domain stability analysis method for grid-connected inverter with PR control based on floquet theory. *IEEE Trans. Ind. Electron.* **68**, 11125-11134 (2021).

9. Ye, T. *et al.* Analysis, design, and implementation of a quasi-proportional-resonant controller for a multifunctional capacitive-coupling grid-connected inverter. *IEEE Trans. Ind. Appl.* **52**, 4269-4280 (2016).

10. Teodorescu, R., Blaabjerg, F., Liserre, M. & Loh, P. C. Proportional-resonant controllers and filters for grid-connected voltage-source converters. *IEE Proc.-Electr. Power Appl.* **153**, 750-762 (2006).

11. MacFarlane, A. G. & Postlethwaite, I. The generalized Nyquist stability criterion and multivariable root loci. *Int. J. Control* **25**, 81-127 (1977).

12. Smith SW. The scientist and engineer's guide to digital signal processing (California Technical Publishing, San Diego, CA, 1999).

13. Hagiwara, T. & Araki, M. On preservation of strong stabilizability under sampling. *IEEE Trans. Autom. Contr.*, **33**, 1080-1082 (1988).

14. Xiong, J. & Lam, J. Stabilization of networked control systems with a logic ZOH. *IEEE Trans. Automat. Control* **54**, 358-363 (2009).

15. Loncar, J., Hrabar, S., & Muha, D. Stability of simple lumped-distributed networks with negative capacitors. *IEEE Trans. Antennas Propag.* **65**, 390-395 (2016).

16. Kuo, B. C. Digital Control Systems. (Oxford Univ. Press, Oxford, 1995).

17. Vasquez, J. C. *et al.* Modeling, analysis, and design of stationary-reference-frame droop-controlled parallel three-phase voltage source inverters. *IEEE Trans. Ind. Electron.* **60**, 1271-1280 (2012).

18. Aarts, R. M. & Janssen, A. J. On-axis and far-field sound radiation from resilient flat and dome-shaped radiators. *J Acoust. Soc. Am.* **125**, 1444-1455 (2009).

19. Kim, D., Safdari, A. & Kim, K. C. Sound pressure level spectrum analysis by combination of 4D PTV and ANFIS method around automotive side-view mirror models. *Sci. Rep.* **11**, 1-15 (2021).

20. Wang, X., Loh, P. C. & Blaabjerg, F. Stability analysis and controller synthesis for single-loop voltage-controlled VSIs. *IEEE Tran. Power Electron.* 32, 7394-7404 (2017).

21. Kaizer, A. J. Modeling of the nonlinear response of an electrodynamic loudspeaker by a Volterra series expansion. *J. Audio Eng. Soc.* **35**, 421-433 (1987).

22. Volke, A., Wendt, J. & Hornkamp, M. IGBT modules: technologies, driver and applications (Infineon Technologies AG, Munich, 2012).

23. Zhang, Z., Guo, B. & Wang, F. Evaluation of switching loss contributed by parasitic ringing for fast switching wide band-gap devices. *IEEE Trans. Power Electron.* **34**, 9082–9094 (2019).

24. Yang, X., Xu, M., Li, Q., Wang, Z. & He, M. Analytical method for RC snubber optimization design to eliminate switching oscillations of SiC MOSFET. *IEEE Trans. Power Electron.* **37**, 4672–4684 (2022).

25. Cree. Datasheet of SiC MOSFET C3M0060065D. https://assets.wolfspeed.com/uploads/2024/01/Wolfspeed_C3M0060065D_data_sheet.pdf (2024)

26. Hahn, P. Theoretical diversity improvement in multiple frequency shift keying. *IEEE Trans. Commun.* **10**, 177-184 (1962).

27. Kim, J., Park, K. C., Park, J. & Yoon, J. R. Coherence bandwidth effects on underwater image transmission in multipath channel. *Jpn. J. Appl. Phys.* **50**, 07HG05 (2011).

28. Schueckler, J. A. Digital image processing system for slow scan television. Preprint at https://scholarworks.rit.edu/theses/5629 (1981).

29. Neuvo, Y., Cheng-Yu, D. & Mitra, S. Interpolated finite impulse response filters. *IEEE Trans. Acoust., Speech, Signal Processing* **32**, 563-570 (1984).

30. Harris, F. J. On the use of windows for harmonic analysis with the discrete Fourier transform. *Proc. IEEE* **66**, 51-83 (1978).
