# Supplementary material for: Digital non-Foster-inspired electronics for broadband impedance matching
Source: Nat Commun. 2024 May 21;15:4346. doi: 10.1038/s41467-024-48861-6 (PMC11109259; doi:10.1038/s41467-024-48861-6)
Supplement: Supplementary file 4 — Description of Additional Supplementary Files [file 41467_2024_48861_MOESM4_ESM.pdf]

Description of Additional Supplementary Files

Supplementary Movie Legend:

Supplementary Movie 1: This video demonstrates the superiority of the proposed digital non-Foster-inspired electronics in terms of arbitrary tunability, excellent stability and high-power handling. Five-fold bandwidth enhancement can be observed by the proposed electronics , by compared with existing analogue non-Foster methods.
